# Do Preschools Offer Healthy Beverages to Children? A Nationwide Study in Poland

**DOI:** 10.3390/nu9111167

**Published:** 2017-10-26

**Authors:** Joanna Myszkowska-Ryciak, Anna Harton

**Affiliations:** Department of Dietetics, Faculty of Human Nutrition and Consumer Sciences, Warsaw University of Life Sciences (WULS), 159C Nowoursynowska Str., 02-776 Warsaw, Poland; anna_harton@sggw.pl

**Keywords:** preschool, nutrition recommendation, beverages, sugars, dietary prevention, preschool children

## Abstract

Background: Children’s beverage consumption patterns have received increased attention in light of the obesity epidemic in this group. In day care centers (DCCs), children spend up to 10 h a day, and typically consume half to three quarters of their daily food intake. The purpose of the study was to investigate what beverages are typically served to children in preschools in Poland, and to evaluate the practices associated with adding sugar and other sweetening agents to beverages. Methods: Direct interviews with preschools staff were conducted with a questionnaire regarding offered beverages and adding sugar and other sweetening agents. The menu of 10 consecutive days and inventory reports were analyzed to verify information. Results: A total of 720 preschools were included in the study. Cocoa and milk coffee substitute were served in 95% of preschools, followed by compote (92%), tea (84%), fruit/herbal tea (73%) and water (69%). Water was the only beverage available between meals (93% DCCs). 86% of preschools added sugar to tea/cocoa/coffee substitute drinks, and 74% to compote. Conclusions: In the majority of preschools, beverages which are not recommended were offered. Such an assortment of beverages and common practice of sweetening can increase the amount of added sugar in a children diet. Nutrition education and legal regulations concerning the assortment of beverages served in preschools are urgently needed.

## 1. Introduction

The rapid global increase in the prevalence of childhood overweight and obesity indicate an urgent need to identify the factors contributing to this situation [1,2,3]. Adequate energy and nutrients intake are required for children’s growth and development, but excessive energy consumption over the long term can lead to overweight and obesity [4].

Recently, children’s beverage consumption patterns have received increased attention in light of the nation’s and global obesity epidemic in this population group [5,6,7]. Some data indicate that sugar-sweetened beverages (SSB) might significantly contribute to excessive intake of calories due to high amount of added (free) sugars [8]. Briefel et al. [9] found that sugar from sweetened beverages contributed more than 200 extra calories in school-aged children’s daily energy intake. Moreover, children drinking SSB on a regular basis have a 17% to 20% increase in total calorie consumption [10,11]. For many SSBs, sugar is the only or major calorie source, therefore they are less likely to induce satiety compared with ingestion of protein or fat, and thus their consumption may result in inadequate calorie compensation, ultimately adding to the total calories consumed [11,12,13]. De Boer et al. [14] in a large cohort of children followed longitudinally from age 2 to 5 years showed that consuming SSB was associated with higher BMI (body mass index) z-score and/or a greater increase in BMI z-score over time.

Among sugar-sweetened beverages, soft drinks and fruit drinks that are not natural juices, are listed first. But the list is much longer: the term SSBs includes carbonated drinks, fruit drinks, flavored waters, energy drinks, sports drinks, tea, chocolate milks, flavored milk, and coffee [15]. Consequently, by definition beverages containing no added sugars, such as bottled water, 100% fruit juice, milk and non-calorically sweetened beverages are excluded. In 2015 World Health Organization (WHO) reaffirmed the global population recommendation to maintain the intake of so called ‘free’ sugars to fewer than 10% of total energy intake. The WHO report suggested that halving this limit to 5% could have additional health benefits [16]. To achieve this goal, it is necessary not only to limit the intake of sugar and sweets, but also other sources of ‘free’ sugars such as honey, syrups, fruit juices and fruit juice concentrates. Water and milk should be offered to children as healthy beverages.

Beverage preferences and consumption patterns begin to develop early in childhood and can persist over time [17,18,19]. Briefel et al. [9] stated that improvements in beverage selections resulted in a projected savings of 10% of total daily calories and 10.5 teaspoons (48%) of daily added sugars intake over the student population. In Poland more than 1140.6 thousand children (84.2% of children in the age group of 3–5 years) were enrolled in day care centers (DCCs) in 2015–2016 [20]. In DCCs, children spend up to 10 h a day, and typically consume half to three quarters of their daily energy while in full-time child-care programs, demonstrating the potential influence of DCCs on children’s overall diet quality [21,22]. Consuming the right amount of good quality drinks is an integral part of a healthy diet, and might act as a preventive factor for energy oversupply. There are no mandatory guidelines in Poland for DCCS regarding the beverages served to children. These institutions can decide about the quality and quantity of beverages offered to children. Studies show that menus provided by DCCs are not always correct and balanced, and these institutions often commit typical nutritional errors in this age group as a low supply of dairy products, vegetable and fruits with an excessive amount of sweets and sugar [23,24]. However, there are very limited data on the assortment of beverages served to children in DCCs, as well as the sweetening-practices in DCCs. The available studies are for individual institutions, and are usually limited to data on nutrients intakes, not products/food served to children [23]. Taking that into consideration, we decided to focus on the type of beverages served to children in DCCs in Poland. In Poland there are public (state) and non-public (private) institutions. The first are almost entirely financed by the state, while the private ones are maintained solely with tuition fees paid by children attending the facility. Our preliminary observations showed that public DCCs allocate a significantly higher amount of money to children's feeding, which may translate into a different products range. That is why we decided to analyze public and non-public DCCs separately.

The purpose of the study was to investigate what beverages are typically served to children in pre-school settings, as well as to evaluate the practices associated with adding sugar and other sweetening agents to beverages in preschools in Poland.

## 2. Materials and Methods

### 2.1. General Information

**Figure 1:**
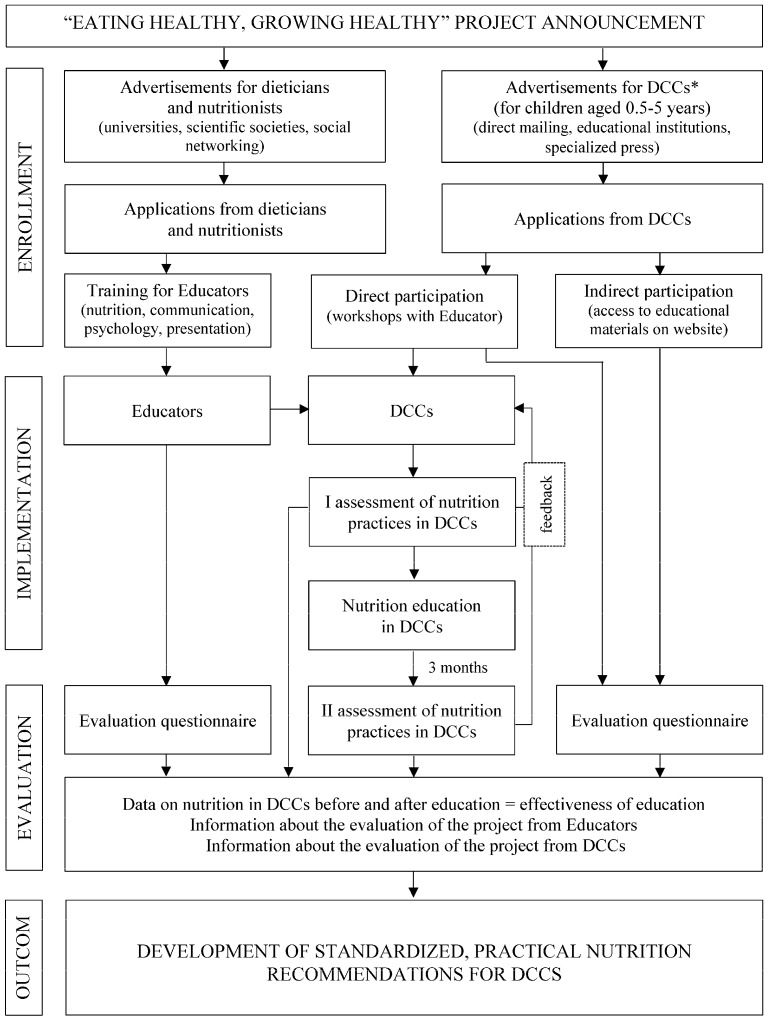
The overview of the project Eating healthy, growing healthy. * DCCs—day care centers.

The presented study is part of a research and education project Eating healthy, growing healthy granted by Danone Ecosystem. The overview of the project is presented in Figure 1. The aim of the project was to improve the nutrition of children in day care centers through the nutritional education of the staff conducted by specially trained educators. The first stage of activities is the analysis of nutrition and nutrition-related practices in the institutions (I assessment). Participation in the project is voluntary and totally free of charge for facilities. The program was intended to cover 2500 DCCs (including nurseries, preschools, and other type of DCCs for children aged 0.5–6 years old) throughout Poland in 2015–2017, and preliminary results from preschools are presented in this paper. Recruiting process takes place via written invitations sent to the institutions after receiving a positive opinion from government educational institutions and local authorities, as well as through a promotional campaign on websites devoted to the education of children.

Institutional Review Board approval was not necessary for this project because it was not deemed human subjects research by the University of Life Sciences Center Institutional Review Board. No personal data were collected about children attending care facilities and institutions staff within the project; applicants (preschools directors) were informed about the purpose and scope of the program, and the possibility of withdrawing from it at any stage without giving any reason.

### 2.2. Study Setting

In this study, we focus only on preschools (attended by children aged 3–5 years old). For the analysis, the preschools with the completed first nutritional analysis were selected, and only these offering full board, i.e., 2 main meals (breakfast, lunch) and 1 or 2 snacks. The analysis excludes childcare facilities working in the part-time system due to a lower number of meals and beverages offered. The above criteria were fulfilled by 720 institutions, and data from all these institutions were analyzed.

### 2.3. Analysis of Beverages Supply

This analysis focused on the types of beverages served with meals or available between meals within 10 consecutive days. The study also included data on the practice of adding sugar and other sweetening agents to served beverages (sweetening practices). Data were obtained by face-to-face interviews with preschools directors and/or staff responsible for nutrition, and by reviewing menus and inventory reports (including information on all foodstuffs purchased by preschool). All interviews were conducted by specially trained interviewers. A questionnaire collected information about the types of typical beverages offered in preschools. After a preliminary analysis of preschool menus, we identified 14 typical beverages for further analysis. They include: water, tea, herbal/fruit tea, compote, juices, fruit drinks, cocoa and coffee substitute with milk, milk and flavored milk, milk tea, water with sugar additives (honey, fruit syrup), water with sugar-free additives (slices of lemon, orange, mint leaves), fruit-milk shakes and liquid jelly/fruit-flavored starch jelly. Some of these drinks are typically Polish, and traditionally served to children in DCCs. Usually they are prepared at the child-care centers from scratch. In traditional recipes, the addition of sucrose (white sugar) is typically suggested. However, because these beverages are prepared by the DCCs, the recipe can be modified for sugar content. The facility may dispense with sweetening of the drink, but it can also replace white sugar with other sweeteners, such as honey, fruit syrup or other sugar (cane, brown). Thus, particular attention has been paid to practices related to sweetening of most frequently served self-prepared beverages. The questionnaire included questions on whether beverages were sweetened and what type of sweetener was used. The nutritional value of these typical Polish beverages was calculated based on typical recipes provided by The National Food and Nutrition Institute [25], in the absence of a recipe in the Institute’s database, information from preschool were taken into account. All calculations were conducted in computer program Energia© v 4.1 (Copyright© 1997/2006 by Andrzej Miegoć, Warsaw, Poland) with Polish Nutrition Database [25]. Milk, yoghurt or kefir served with cereals were not considered a beverage. In addition, 10-day menu and inventory reports from 10 consecutive days were analyzed to verify obtained information. In total, 7200 daily menus and 7200 daily inventory reports were examined. Based on collected menus, we checked whether the beverages declared by the preschool in the questionnaire were present at least once during 10 consecutive days. If the beverage declared by preschool in the questionnaire was not listed in the 10 days menu, we did not take this data for further analysis due to the occasional occurrence. Inventory reports allow us to estimate the amount of beverages per child (data not shown in this article).

### 2.4. Statistical Analysis

We used Statistica version 12 (Copyright©StatSoft, Inc., 1984–2014, StatSoft Polska Sp. z o.o., Cracow, Poland) for all statistical analyses. For the general characteristic of preschools participating in the study: number of children, the amount of money allocated per day to feed a child (financial rate) means, standard deviations (SD), and medians were calculated. Data on beverages supply were analyzed in total group (all preschools) and according to type of preschools: public vs. non-public. Statistical significances for qualitative variables were determined using Pearson’s chi-square test. In the case of quantitative variables, the Shapiro-Wilk statistic for testing normality was used. Due to lack of normal distribution of quantitate data, U Mann–Whitney test was used to check the significant differences. The differences were considered significant at *p* ≤ 0.05.

## 3. Results

### 3.1. General Characteristics of Preschools

The study involved 720 preschools from the total number of 11,331 institutions in Poland [20] (6.35% of all preschools in the country). Most of these were public facilities with a higher average number of children per institution, and a lower average amount of money allocated per day to feed a child (Table 1). In total, 79,135 children aged 3–5 attended in all examined institutions in studied period.

### 3.2. Characteristics of Beverages Offered in Preschools

The preschools reported offering a variety of beverages to the children. Some of them are commonly available on market beverages, of known composition and nutritional value (e.g., tea, herbal/fruit tea, milk, cocoa milk, yoghurt, kefir, juices, fruit drinks or water). On the other hand, kindergartens also served self-prepared typical Polish beverages: compote (beverage made of typical Polish fruits as apples, strawberries, cherries, currants boiled in large amount of water, milk coffee substitute (usually wheat; prepared with milk instead of water), milk tea (called in Polish ‘bawarka’; prepared with large amount of milk instead of water), fruit-milk shakes (milk mixed with typical Polish fruits, e.g., strawberries), and liquid fruit-flavored jelly/starch jelly (water with fruit flavor additives or fruit syrup, and same gelatin or starch for texture). Since these drinks are not widely known outside of Poland, and they provide significant amounts of energy (as they may contain nutrients such as sugars, protein, fat), their characteristics are shown in Table 2. Traditional recipes usually suggest a large amount of added sugar for a sweet taste. Typically, milk coffee substitute (made from roasted barley, beetroot and chicory), and milk tea are served for breakfast. Fruit-milk shakes are often provide with snacks, whereas compote, liquid fruit-flavored jelly and starch jelly—with lunch (traditionally in Poland it is the main meal, and usually provides about 30% of total daily energy). Preschools also provided children with water with flavors additives: honey or fruit syrup or slices of fresh fruit or fresh herbs (e.g., mint). In the case of the former, the energy value of 100 mL of the beverage is about 20 kcal (provided by sugars), in the latter it can be omitted due to its very low value. Other beverages as tea, herbal/fruit tea, water, juices, fruit drinks, and fermented milk beverages are popular also outside of Poland, and they have typical nutritional value [25,26].

### 3.3. Beverages Typically Served in Preschools

Table 3 presents the types of beverages typically served in the preschools, overall, and by public and non-public preschools. More than 90% of preschools typically offered to children cocoa, wheat coffee substitute and compote. Fewer non-public preschools declared such practices. Tea was served in 84% establishments, while fruit/herbal tea in 73%. More private establishments declared fruit/herbal tea in menus, but the result was not statistically significant (tendency). Water for meals was provided by 69% of preschools, but in 93% it was available for children between meals. Nearly half of the preschools served 100% fruit juices to children, and significant differences were also observed. About 1/5 of the preschools offered to children fruit drinks, whereas other drinks were served in less than 10% of facilities.

### 3.4. Sweetening-Related Practices in Preschools

Some of the preschools did not add any sugar for served beverages. In the case of tea, herbal/fruit tea, milk tea, cocoa and milk coffee substitute only 14% of the preschools declared no added sweeteners (9% of public and 25% of non-public). The compote was not sweetened in 26% of preschools (no significant difference depending on the type of preschools). Sugar or honey were most commonly used to sweeten, and significant differences between types of preschools were observed (Table 4). Sugar was more often used in non-public institutions, while honey in the public. Syrups or other types of sugar (brown, cane) have been used infrequently, both in public and non-public institutions.

## 4. Discussion

The prevalence of childhood obesity is increasing across European countries [27]. Compared to other European countries, the incidence of overweight and obesity in children in Poland is moderate. In the pre-school children survey conducted in 2008 in the Rzeszow region, 9.1% of girls and 9.9% of boys were overweight, whereas in 7.2% of girls and 8.4% of boys obesity was noted [28]. However the situation is changing in Poland: recent data from COSI study (European Childhood Obesity Surveillance Initiative) indicated that one out of every three eight-year-olds has excessive weight [29]. Evidence indicates that a large proportion of children who have obesity before puberty can develop obesity in early adulthood, and physiological and psychological health consequences during childhood can continue into adolescence and adulthood [30,31,32].

Some modifiable risk factors for obesity include dietary and physical activity behaviours, which to a large extent, are learned at an early age [33,34]. The food and beverages offered at daycares might play a very important role in the formation of preschoolers’ eating habits. Thus, child care settings may contribute to healthy beverage consumption patterns, and in that way lower the risk of excessive body mass.

In our study, the majority of preschools (over 90%) customarily served cocoa/milk coffee substitute and compote to children. These beverages, according to the recipe, contain significant amounts of sugar. Compote, due to main ingredient (fruit) contains mainly simple sugars (added and naturally occurring in fruits) and some amounts of vitamin C. In the children's population in Poland, low intake of this vitamin is noted, especially in the group of preschoolers [35,36]. However, the content of vitamin C during thermal processing is significantly reduced (more than 50%), so the compote, opposite to fresh fruit or natural 100% fruit juices, is not a good source of it [37]. Therefore, compote does not improve the quality of children's diet, but rather contributes to increased consumption of simple and added sugars. This is particularly evident in the case of added sugars: because about 3/4 of the establishments typically serve the compote sweetened with different types of caloric sweeteners (sugar, honey, syrups).

Cocoa and milk coffee substitute offered in the majority of preschools, according to the recipe, are prepared with milk. Milk is a very important component of children’s diet, and plays a vital role in meeting nutrient intake recommendations [38]. Several European Union (EU) Member States (France, Belgium, Ireland, Spain) recommend around 3–4 servings of dairy products per day for children. Others (Denmark, Finland, The Netherlands, Poland) recommend around 500–600 mL dairy foods per day for children. However, many children fail to meet the dietary recommendations for dairy intake and hence nutrient requirements [36,39]. In a study of beverage intake during middle childhood, milk consumption among girls almost always or always served milk at meals and snacks was two times higher than it was for girls rarely or never served milk [40]. Thus, milk and dairy are recommended to be part of breakfast in childcare facilities [41]. However, special attention should be paid to the flavored milk, as well as chocolate/cocoa/flavored milk or milk shakes. These beverages contain not only flavors, but also significant amounts of added sugar. Due to the sweet taste, they are often preferred by children in comparison to plain milk. Briefel et al. noted that flavored milks accounted for a student population mean of 76 daily calories and mean of 1.5 teaspoons of added sugars [9]. Unfortunately, our study confirms this unfavorable trend: 95% of the preschools served milk with additives, and at the same time 92% of preschools did not offered plain milk as a beverage for children. Moreover, in the case of self-prepared beverages (usually cocoa milk and always milk coffee substitute), the staff of facility can decide about added sugar. However, only 14% of all kindergartens did not practice sweetening. These are unfavorable practices that can also contribute to an increase in the consumption of added sugars by pre-school children.

A large proportion of examined kindergartens serve children tea and fruit/herbal tea. Beverages such as green tea and black tea (also natural coffee or energy drinks) are not recommended for preschoolers. Some of them contain not only large amounts of theine and/or caffeine (coffee, tea, energy drinks), but also substances that can reduce the availability of iron from the diet [42]. Black tea was found almost twice as inhibiting as green tea or peppermint tea and over three times as inhibiting as herbal tea [43]. In particular, drinking tea with meals should be avoided as it is likely to inhibit non-heme iron absorption at-risk groups including children under 6 years of age. In examined kindergartens, tea was usually served to meals, whereas between meals children had access to water. Watt et al. [44] focused on the nutritional impact of drinks consumption in pre-school children. The authors found that the diets of tea drinkers were lower in iron and vitamin C than those of non-tea drinkers. In Poland, low intake of iron in children is observed: 90% of 3 years old children did not meet the recommended intake for this nutrient [45]. The high prevalence of pre-school settings, which commonly serve tea, can have a negative effect on the children’s iron status. It is worth to mentioned, that a small percentage (3%) of preschools declared offering of so-called milk tea. Such milk tea was a popular beverage in Poland in the past years when cocoa and coffee were difficult to purchase. The drink formula for children includes a large proportion of milk in ready-to-drink beverage. Adding milk to tea is a beneficial practice as it can enhance iron absorption (16%) [43]. However, it is definitely an unfavorable practice to sweetened these type of beverages: in three out of four preschools such practices were noted.

Fruit juices in public opinion are recognized as products of high nutritional value, specially good source of vitamins (mainly vitamin C). Due to the sweet taste they are preferred by children: children 2 to 18 years of age consume nearly half of their fruit intake as juice, which lacks dietary fiber and predisposes to excessive caloric intake [46]. Fruit juice offers no nutritional advantage over whole fruit, but in preschool settings might be more convenient to served (no preparation). Moreover, because juice is viewed as nutritious and healthy, limits on consumption are not usually set by parents [47]. The American Academy of Pediatrics recommends no more than four to six ounces (115–170 mL) a day of fruit juice for children four- to six-years old [48]. However, it should be stressed that these recommendations apply only to 100% natural sugar-free juices. Studies point to frequent over-supply of juices in pre-school settings [49,50,51]. In present study 100% natural juices were served by less than half of preschools, whereas one out of four of facilities offered fruit drinks. Although fruit juices are a good source of vitamin C, due to the high content of free sugars they should not be the first choice beverage in preschools. It is worth stressing that juices are more expensive compared to other beverages. In our study, more non-public institutions served them to children, which could have been influenced by a higher food rate per child in these DCCs.

Water is an essential nutrient, and has an important role in overall bodily system functioning, and in the prevention of chronic conditions and diseases common in the 21st century (e.g., obesity, dental caries). Is it known, that children are not consuming enough water, instead opting for sugar-sweetened beverages (SSBs), 100% fruit juice, and other beverages [52]. Increasing water consumption may help limit excess weight gain [53], but consuming water instead of SSBs can also prevent dental caries [54,55,56]. Because children spend most of their day in DCCs, ensuring that drinking water is available in these settings is a fundamental public health measure [57]. Increasing drinking water access in preschools is a step in the right direction toward encouraging children water intake. In our study, the majority of preschools (93%) provided children with free access to water between meals. Identical results were obtained by Sisson et al. [58] in a study of 314 kindergartens from Oklahoma State, US. However, serving water as a beverage to meals was declared less frequent in our study (69%). A small percentage of preschools offered water with flavors and/or sweeteners. Adding fresh fruit or leaves of herbs can make the taste of water more attractive to children, and increase its consumption. However, the addition of caloric sweeteners is not conducive to shaping proper eating habits, and should not be practice in care settings.

The study clearly demonstrates cultural differences in the supply of beverages in preschools. In one out of 100 outlets, children were given a liquid fruit jelly or a liquid starch fruit jelly, which is unique for Poland. These drinks are characterized by a low nutritional value: do not contain any important nutrients except for added sugar and flavors/colors additives. However, because of the sweet taste and attractive color they are eagerly consumed by young children. In the past years they were often served as a cheaper alternatives to compote in Poland. However, taking into account current dietary recommendations on sugar intake [16], they should not be offered as beverages in care settings.

Based on the actual recommendations for care setting, the best choices for children’s drinks are water and nonfat or low-fat milk (soya alternatives), and (optionally) 100% fruit juices in limited amount [59,60]. As children consume one half to two thirds of their daily nutrition requirements at full-time child-care programs (8 or more hours/day) [61], menus designed at daycares might play a very important role in the formation of preschoolers’ eating habits. In our study, majority of the preschools did not meet the recommendation for proper beverage selection in the preschool menu, except of water availability. Our findings are consistent with the observations of Benjamin Neelon et al. [62], who similarly observed prevalent supply of high-energy beverages in DCCs in Mexico.

The present study provides a unique insight into the nutritional quality of beverages typically served to young children in child-care centers in Poland, however some limitations should be stressed. The study is particularly focused on beverages supply and sweetening practices on institutions level. Due to large sample of preschools, we did not assess actual dietary intake in children. However, study by Ball et al. [63] in the USA suggests that children consume 50–100% of what they are offered in child-care centers. It can be expected that improving the nutritional quality and quantity of beverages offered in DCCs may affect intake, but it was not the aim of the present study. Future studies should explore dietary intake to assess what children actually consume in child care throughout Poland.

A major strength of this study is the large sample of preschools from whole Poland, including public and non-public (however, the sample selection was not random). The method of data obtaining (face-to-face interviews led by trained interviewers using a validated questionnaire), and verification of data based on 10-day menus and inventory reports are also an advantage.

## 5. Conclusions

In the face of an epidemic of obesity, healthy beverages in adequate amount should be available and accessible to children in all preschools settings. Optimizing the assortment of beverages offered to children in DCCs might reduce the intake of added sugars in preschoolers. Future studies should determine barriers (e.g., lack of knowledge, luck of founds) to fulfill the dietary recommendations in child-care centers and realistic minor changes that can improve nutrition quality of beverages.

## Figures and Tables

**Table 1:** General characteristics of preschools: number of children and costs of one day menu per capita.

Characteristic of Institutions	Public Preschools *n* = 529	Non-Public Preschools *n* = 191	Total *n* = 720
Number of children:			
Mean ± SD	123 ± 54 ^1^	74 ± 59	110 ± 59
Median/Min/Max	123/9/411	55/10/415	108/9/415
Financial rate per 1 child/day/PLN ^2^:			
Mean ± SD	5.8 ± 1.3 ^1^	8.3 ± 2.1	6.4 ± 1.9
Median/Min/Max	5.5/3.0/11.0	8.0/4.0/16.0	6.0/3.0/16.0

^1^ Significant differences between public and non-public preschools, U Mann-Whitney test; ^2^ 1 PLN (Polish zloty) = ~0.23 Euro.

**Table 2:** The average nutritional value of traditional Polish beverages served in preschools.

Beverages (100 mL)	Energy (kcal)	Carbohydrates (g)	Sucrose (g)	Protein (g)	Fat (g)	Dietary Fibre (g)
Compote	69	16.9	14.2	0.2	0.1	0.3
Fruit-milk shakes	109	24.2	1.2	0.7	21.3	0.7
Liquid fruit-flavored jelly	31	5.0	5.0	2.5	0.0	0.0
Liquid fruit-flavored starch jelly	49	12.9	8.6	0.2	0.1	1.0
Milk coffee substitute (wheat)	90	12.2	5.7	3.2	2.9	0.6
Milk tea	51	7.4	5.0	1.7	1.6	0.0

**Table 3:** Beverages offered with meals to children in participating preschools: comparison of public and non-public institution, and data for the total group.

Beverages	Public Preschools *n* = 529 (%)	Non-Public Preschools *n* = 191 (%)	*p*-Value Public vs. Non-Public	Total *n* = 720 (%)
Cocoa and milk coffee substitute: yes	516 (98%)	166 (87%)	*p* = 0.0000 ^1^	682 (95%)
Compote: yes	496 (94%)	163 (85%)	*p* = 0.0003 ^1^	659 (92%)
Tea (black, green): yes	448 (85%)	157 (82%)	*p* = 0.4208	605 (84%)
Herbal/fruit tea: yes	379 (72%)	149 (78%)	*p* = 0.0881	528 (73%)
Pure water: yes	371 (70%)	123 (64%)	*p* = 0.1432	494 (69%)
Juices 100% natural: yes	246 (47%)	72 (38%)	*p* = 0.0356 ^1^	318 (44%)
Fruit drinks (not 100% juices): yes	129 (24%)	43 (23%)	*p* = 0.6029	172 (24%)
Milk: yes	34 (6%)	21 (10%)	*p* = 0.0416 ^1^	55 (8%)
Water with honey or fruit syrups: yes	31 (6%)	12 (6%)	*p* = 0.8326	43 (6%)
Fruit-milk shakes: yes	20 (4%)	8 (4%)	*p* = 0.8027	28 (4%)
Milk tea (black): yes	17 (3%)	5 (3%)	*p* = 0.6817	22 (3%)
Water with fresh lemon/orange/mint: yes	11 (2%)	5 (3%)	*p* = 0.6652	16 (2%)
Fermented milk beverages (kefir, yoghurt): yes	6 (1%)	1 (1%)	*p* = 0.4609	6 (1%)
Liquid jelly/fruit starch jelly: yes	3 (1%)	3 (2%)	*p* = 0.1909	6 (1%)
Water available between meals: yes	491 (93%)	179 (94%)	*p* = 0.6747	670 (93%)

^1^ Significant differences between public vs. non-public preschools, chi^2^ Pearson test.

**Table 4:** Sweetening of self-prepared beverages offered with meals to children in participating preschools: comparison of public and non-public institution, and data for the total group.

Beverages/Sweetener Used	Public Preschools *n* = 529 (%)	Non-Public Preschools *n* = 191 (%)	*p*-Value Public vs. Non-Public	Total *n* = 720 (%)
Tea, herbal/fruit tea, milk tea, cocoa and milk coffee substitute:				
sugar (white)	125/(24%)	65/(34%)	0.0051 ^1^	190/(26%)
sugar (cane, brown)	14/(3%)	7/(4%)	0.4734	21/(3%)
honey	335/(63%)	65/(34%)	0.0000 ^1^	400/(56%)
fruit syrups	5/(1%)	6/(3%)	0.0267 ^1^	11/(1%)
no added sugars	50/(9%)	48/(25%)	0.0000 ^1^	98/(14%)
Compote ^2^:				
sugar (white)	184/(37%)	81/(50%)	0.0044 ^1^	265/(40%)
sugar (cane, brown)	19/(4%)	7/(4%)	0.7918	26/(4%)
honey	143/(29%)	28/(17%)	0.0032 ^1^	171/(26%)
fruit syrups	24/(5%)	2/(1%)	0.0398 ^1^	26/(4%)
no added sugars	126/(25%)	45/(28%)	0.5775	171/(26%)

^1^ Significant differences between public and non-public preschools, chi^2^ Pearson test; ^2^ compote was served in 496 public and 163 non-public preschools.
